# Criteria for Applying the Lucas-Washburn Law

**DOI:** 10.1038/srep14085

**Published:** 2015-09-14

**Authors:** Kewen Li, Danfeng Zhang, Huiyuan Bian, Chao Meng, Yanan Yang

**Affiliations:** 1 China University of Geosciences (Beijing), 29 Xueyuan Road, Beijing 100083, China; 2 Stanford University, 367 Panama St., CA 94305, USA

## Abstract

Spontaneous imbibition happens in many natural and chemical engineering processes in which the mean advancing front usually follows Lucas-Washburn’s law. However it has been found that the scaling law does not apply in many cases. There have been few criteria to determine under what conditions the Washburn law works. The effect of gravity on spontaneous imbibition in porous media was investigated both theoretically and experimentally. The mathematical model derived analytically was used to calculate the imbibition rates in porous media with different permeabilities. The results demonstrated that the effect of gravity on spontaneous imbibition was governed by the hydraulic conductivity of the porous media (permeability of the imbibition systems). The criteria for applying the Lucas-Washburn law have been proposed. The effect of gravity becomes more apparent with the increase in permeability or with the decrease in CGR number (the ratio of capillary pressure to gravity forces) and may be ignored when the CGR number is less than a specific value 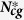 ≅ 3.0. The effect of gravity on imbibition in porous media can be modeled theoretically. It may not be necessary to conduct spontaneous imbibition experiments horizontally in order to exclude the effect of gravity, as has been done previously.

Spontaneous imbibition describes the phenomenon of a wetting phase fluid invading into a porous medium and displacing a non-wetting resident phase at a constant external pressure. This process exists in many industries and plays an important role in numerous natural, chemical, and commercial processes^1^,^2^,^3^,^4^,^5^,^6^,^7^,^8^,^9^,^10^,^11^,^12^,^13^,^14^,^15^,^16^,^17^,^18^,^19^,^20^,^21^,^22^,^23^,^24^,^25^. Spontaneous imbibition is of great significance not only for its fundamental aspects but also for its technological applications like liquid delivery in nano materials (liquid imbibition into nanotubes), filtration (liquid imbibition into porous material), construction (water penetration into concrete or cement pastes), printing processes (ink penetration in paper or coating of paper), irrigation (displacement of gas by water), and oil recovery (displacement of gas or oil by a different liquid)^4^,^5^,^6^,^7^,^8^,^9^,^10^,^11^,^12^,^13^,^14^,^15^,^16^,^17^,^18^,^19^.

It has long been known that the mean advancing front follows Washburn’s law, <h> ~ t^1/2^ in many cases^1^. Later on, similar models have been reported in different forms^11^,^12^,^13^,^14^. Actually the behavior of <h> ~ t^1/2^ has been even earlier reported by Lucas *et al.*^25^. Therefore, the behavior of <h> ~ t^1/2^ was termed as Lucas-Washburn’s law in the following parts.

There is widespread literature on the wetting dynamics of homogeneous and structured surfaces^1^,^2^,^3^,^4^,^5^,^6^,^7^,^8^,^9^,^10^,^11^,^12^,^13^,^14^,^15^,^16^. Soriano *et al.*^5^ reported experiments on spontaneous imbibition of a viscous fluid by a model porous medium in the absence of gravity. They concluded that the average position of the interface satisfied Lucas-Washburn’s law. Scaling of the interface fluctuations suggested a dynamic exponent of about 3.0, indicative of global dynamics driven by capillary forces.

Recently Xue *et al.*^21^ showed for aqueous electrolyte imbibition in nanoporous gold that the fluid flow could be reversibly switched on and off through electric potential control of the solid–liquid interfacial tension. They found that Lucas-Washburn’s law also works in most of these special imbibition processes.

The results from the literature show that Lucas-Washburn-like scaling is not always observed in many spontaneous imbibition experiments because of a lot of factors^4^,^5^,^11^,^12^,^13^. These include non-Newtonian character of the liquid such as ink, the change in the property of the porous media (for example, swelling of the paper fibers during imbibition). Supple and Quirke^4^ have carried out molecular dynamics simulations of nanotubes imbibing oil at an oil/vapor interface at 298 K. Supple and Quirke^4^ found that the imbibition into nanotubes did not obey the macroscopic Lucas-Washburn equation and was very rapid via complex imbibition dynamics. The penetration length was a linear function of time instead of time to the one-half power as foreseen by Lucas-Washburn’s law.

Miranda *et al.*^6^ investigated the behavior of the spontaneous imbibition interface (ink-paper) as a function of time and paper orientation. The results showed that the dynamics of the rough interface depend on the orientation of the paper fibers. However the mechanism behind this observation has not been studied. The results reported by Miranda *et al.*^6^ indicated that the ink-paper interface do not move according to the Lucas-Washburn law.

According to the literature^22^,^23^,^24^, three characteristic regimes may be distinguished for spontaneous imbibition in a single cylindrical tube. Regime I is the inertial regime that is the very beginning of the invasion process, where the liquid invades in a rather ballistic manner into the pore space. In this regime the height of the front scales linearly with the elapsed time t. The duration of this time can be estimated by the scaling model^22^. Regime II is the Lucas-Washburn regime with the “classic” square-root-of-time-behavior (Lucas-Washburn-law), where capillary and viscous forces prevail. Regime III is the regime that is usually the very late period of spontaneous imbibition where gravitational forces significantly affect the dynamics.

Note that both capillarity and gravity forces exist in spontaneous imbibition, although capillarity forces dominate in some cases. Clearly, gravity is not included in Lucas-Washburn’s law, and may be one of the reasons why Lucas-Washburn’s law does not work in some cases^11^,^12^,^13^.

Researchers have tried hard to perform horizontal spontaneous imbibition tests^5^. Even so, few experiments have been conducted in horizontal directions in order to avoid the effect of gravity on spontaneous imbibition^5^. One can see that it is of great significance to investigate the effect of gravity on spontaneous imbibition. However little is known about at what conditions gravity force could be ignored.

In this paper, the criteria for applying the Lucas-Washburn law have been developed. The specific value of CGR number 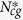 was determined to analyze when the effect of gravity force on spontaneous imbibition can be neglected. It has been proved in this article that the effect of gravity on imbibition in porous media can be considered mathematically. Therefore, it may be unnecessary to conduct spontaneous imbibition experiments horizontally in order to exclude the effect of gravity, as done in the past^5^.

## Theoretical Background

Lucas-Washburn-like models are frequently utilized to investigate spontaneous imbibition. However these equations may not be applied to the cases where gravity is considered. To this end, a linear correlation between the spontaneous imbibition rate and the reciprocal of the cumulative imbibition or recovery, with gravity forces included, was used^13^:


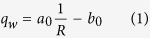


where *R* is the recovery in the unit of pore volume. Note that recovery is the ratio of water imbibed to the pore volume of the porous medium (core sample), directly proportional to imbibition front height, <*h*>, in many cases. *q*_*w*_ is the volumetric spontaneous imbibition rate of the wetting phase and is equal to *dR*/*dt*, directly proportional to the derivative of *h* to imbibition time, *dh*/*dt*.

*a*_0_ and *b*_0_ are two constants associated with capillary and gravity forces, respectively, and are expressed as follows:


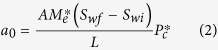


where *A* is the cross-sectional area of the rock sample, *S*_*wf*_ is the water saturation (volume of water divided by the total pore volume of the porous medium) behind the imbibition front, *S*_*wi*_ is the initial wetting-phase (water in this study) saturation in the core sample, 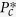 is the capillary pressure at *S*_*wf*_. *L* is the length of the core sample.


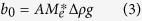


here Δ*ρ* is the density difference between the wetting phase and the nonwetting phase (= *ρ*_*w*_ − *ρ*_*nw*_), *g* is the gravity constant, 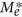 is defined as the global mobility of the two phases.

Obviously equation (1) is reduced to Lucas-Washburn-like model, <*R*> ∼ *t*^1/2^, if gravity force is ignored.

For co-current spontaneous imbibition (the flowing direction of the wetting phase is the same as that of the nonwetting phase), the expression of the effective mobility is represented as follows:


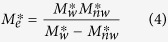


where 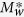 is the wetting phase mobility at *S*_*wf*_ and 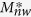 the nonwetting phase mobility at 1−*S*_*wf*_. The wetting and nonwetting phase mobilities are expressed as follows:


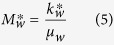



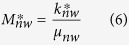


where k^*^_w_ and 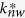 are the effective permeabilities of the wetting and nonwetting phases at *S*_*wf*_ and 1−*S*_*wf*_ respectively, *μ*_*w*_ and *μ*_*nw*_ are the viscosities of the wetting and nonwetting phases.

In the case of gas-liquid two phase flow, equations (1) and (4) can be reduced because the gas mobility is much greater than the liquid mobility^11^,^12^. For gas-liquid two phase flow, the expression of the relationship between imbibition rate and the reciprocal of recovery stays the same as equation (1) but those of *a*_0_ and *b*_0_ are simplified as follows:


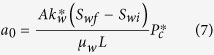



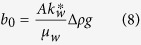


Based on equations (7) and (8), capillary pressure can be calculated:


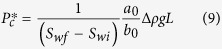


According to equation (10), the effective water permeability at the water saturation of *S*_*wf*_ can be computed as follows:


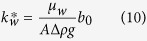


The values of *a*_0_ and *b*_0_ in equations (9) and (10) can be determined from the plot of imbibition rate and the reciprocal of the gas recovery. *S*_*wf*_ can be measured during the water imbibition tests. Therefore, one can infer both capillary pressure and effective water permeability from the experimental data of spontaneous water imbibition using equations (9) and (10).

A great challenge in characterizing spontaneous imbibition behavior in gas-liquid systems was to calculate the effective water permeability *k*_*w*_ and capillary pressure *P*_*c*_ separately. The method (eqs. (9) and (10)) described here may provide a solution to this problem and is especially useful for the porous media with very low permeability such as shale rocks.

Equations (1) and (6) have been used and verified in many cases, including the prediction of oil production^11^,^12^,^13^, scaling of experimental data^17^,^18^. Hognesen *et al.* (2004) analyzed the conditions of the applicability of equation (1). They reported that whether and when one could expect the parameter “*c”* in equation (1) to be constant. Hognesen *et al.* (2004) also showed that the data have a realistic error scatter around the regression curves and the regions where equation (1) is not valid are identified.

For simplification and generalization, equation (1) can also be expressed as follows:


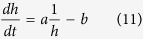


Where *a* and *b* are constants related to *a*_0_ and *b*_0_.

## Results

**Figure 1:**
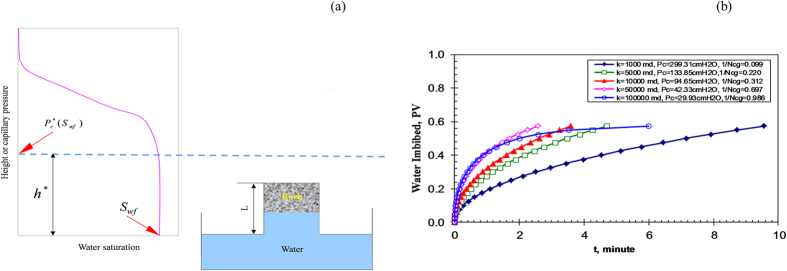
Relationship between water imbibed and time (model results), (a) Schematic of the modeled spontaneous imbibition tests; (b) Model data of spontaneous imbibition.

In this Paper, we studied the effect of gravity on spontaneous imbibition rate in porous media with different properties using equation (1) and experimental data. According to equation (1), the effect of gravity on spontaneous imbibition was governed by the ratio of capillary pressure to gravity forces, or the CGR number (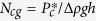) of the imbibition systems. Note that *N*_*cg*_ is a constant. This is because gravity is constant and the capillary pressure is the specific one, 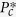, which is also constant (see Fig. 1a)^11^.

The rock (or porous media) with greater permeability generally has smaller capillary forces, the vice versa. Therefore the effect of gravity increases with the increase in permeability or decreases with the increase in capillary number. Theoretically (foreseen by eq. (1)) gravity forces may be ignored when the permeability or CGR number is less than a specific value (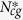). This prediction will be tested in the following section. Note that the value of 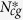 has not been reported before.

Theoretical calculations using equation (1) were based on spontaneous water imbibition into dry (gas-saturated) rocks at a temperature of 20 °C to obtain the specific value of *N*_*cg*_. Rock samples were assumed to position vertically and were contacted with water at the bottom (see Fig. 1a). The values of the rock and fluid property are listed as follows: the diameter of rock (*D*) was 3.40 cm for calculating *A*, the length was 29.5 cm, porosity of the rock (*ϕ*) was 0.386, *S*_*wf*_ = 0.575, *S*_*wi*_ = 0.0, the surface tension of water and contact angle used to calculate capillary pressure were 72.696 dyne/cm and zero respectively, the viscosity of water was 1.0 cp, the relative permeability of water was 0.614, and the permeability of rock samples ranged from 1000 to 100000 md. It is assumed in this study that the length of the rock sample is less than the height corresponding to 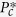, that is, *L* < h^*^ (see Fig. 1a).

The theoretical data of water spontaneous imbibition into gas-saturated rocks were calculated with the above parameters using equation (1) for core samples with different permeabilities. The results are plotted in Fig. 1b as the relationship between water imbibed and time.

One can see the significant effect of permeability or CGR number on spontaneous imbibition, as shown in Fig. 1b. However it is almost impossible to tell the effect of gravity. For this reason, Lucas-Washburn-like model was used to process the theoretical data.

**Figure 2:**
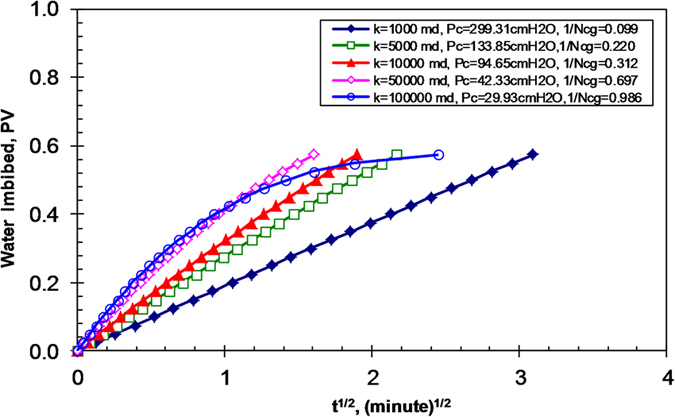
Relationship between water imbibed and the square root of time (model results).

Figure 2 shows the relationship between water imbibed and time to the one-half power using data from Fig. 1. All of the relationships should be linear if Lucas-Washburn’s law applies. One can see that the relationships are not linear once the permeability is greater or the value of 1/*N*_*cg*_ is less than a specific value (gravity forces become dominating).

It is known that the effect of gravity is greater in the core samples with greater permeabilities or capillary number, *N*_*cg*_. But the critical value of *N*_*cg*_, 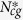, is not known for possibly ignoring gravity force. According to the results shown in Fig. 2, the value of 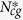 may be equal to 3.0 in the cases studied. This implies that the effect of gravity force on spontaneous imbibition may be neglected and Lucas-Washburn model works if the value of *N*_*cg*_ is greater than 3.0 approximately, and vice versa.

**Figure 3:**
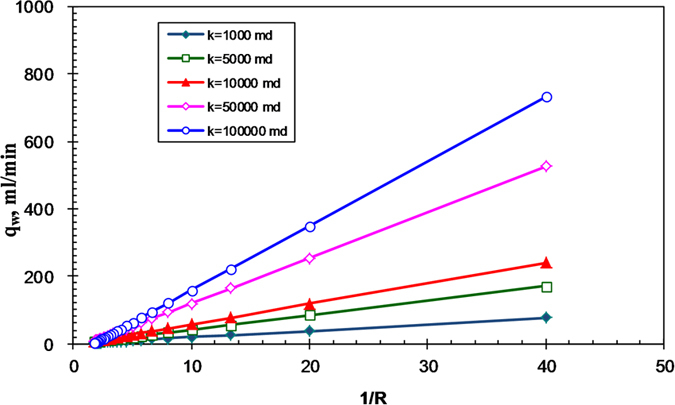
Relationship between water imbibition rate and the reciprocal of recovery (model results).

The same set of data shown in Fig. 2 was used but plotted in a different way by using equation (1) in which gravity force is included. The relationship between the imbibition rate and the reciprocal of recovery is shown in Fig. 3. All of the relationships are linear for the rocks with different permeabilities as expected.

**Figure 4:**
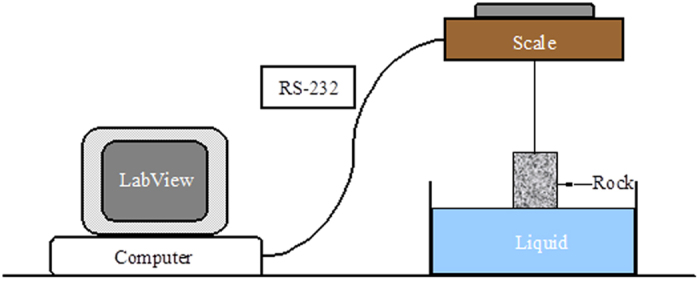
Schematic of apparatus for spontaneous imbibition tests 11.

In the above section, it is demonstrated using the data from the analytical model^11^ verified previously that Lucas-Washburn’s law does not work in the cases in which the gravity force is dominating (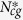 < 3.0). We will verify the above model phenomenon experimentally in the following section. To this end, spontaneous water imbibition experiments were conducted in different types of dry rocks positioned vertically^11^,^12^. The schematic of the apparatus for conducting spontaneous imbibition tests is shown in Fig. 4. The rock sample was hung under a balance which had an accuracy of 0.01 g and a range from 0 to 1600 g. The water imbibed into the core sample was recorded in time by the balance using an under-weighing method and the real-time data were measured continuously by a computer through an RS-232 interface. The purpose of using the under-weighing method was to reduce the experimental error caused by water evaporation. Air was used as the gas phase and distilled water as the liquid phase. The experimental details can be found more in the refs 11, 12, 13.

**Figure 5:**
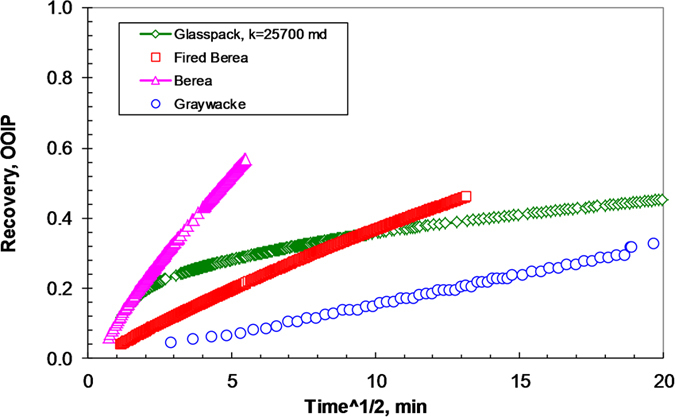
Relationship between water imbibed and the square root of time in different rocks.

Figure 5 shows the relationship between water imbibed and the square root of time in rocks with different permeability, ranging from 0.56 (the greywacke) to over 25700 md (the glass-bead pack). The fired and natural Berea sandstone core samples had permeabilities of 1200 and 804 md respectively. It is obvious that the recovery (directly proportional to imbibition front height) in the glass-bead pack with a high permeability of about 25700 md does not scale with the square root of time, as predicted by Lucas-Washburn’s law. One can see from all of the experimental results, similar to the model data as shown in Fig. 2, that Lucas-Washburn’s law does not apply in cases where the permeability is greater than a specific value.

**Figure 6:**
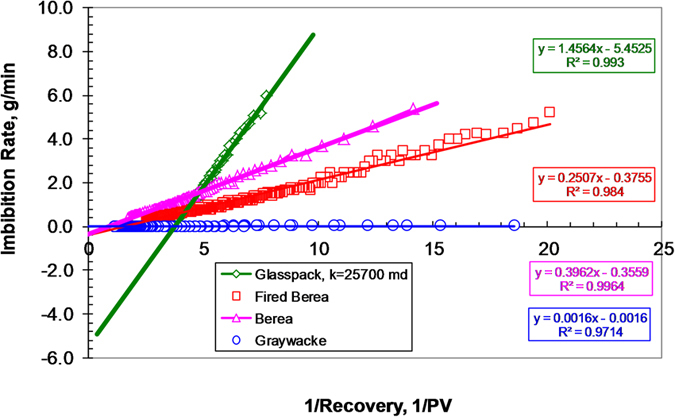
Relationship between water imbibition rate and the reciprocal of recovery in different rocks.

To test equation (1), the experimental data shown in Fig. 5 are plotted in a different way as imbibition rate vs. 1/R and the results are demonstrated in Fig. 6. Remarkably all of the relationships between water imbibition rate and the reciprocal of recovery in very different rocks are linear, as predicted by equation (1). This demonstrates that gravity forces may be well considered in equation (1). The model (eq. (1)) is derived from physical principles and matches the experimental data of spontaneous water imbibition very well with great values of regression coefficient (R^2^). Note that Lucas-Washburn’s law cannot match the spontaneous imbibition data in the cases in which gravity forces dominate.

The above experimental data proved that equation (1) could consider the effect of gravity satisfactorily. On the other hand, both the model and experimental data demonstrate that equation (1) can match the spontaneous imbibition data in both cases where Lucas-Washburn’s law works and does not work, which implies equation (1) is more general in terms of characterizing spontaneous imbibition in porous media.

## Discussion

It is well-known that Lucas-Washburn’s law has been extensively utilized in many processes happened in nature and industries for about one hundred years. The importance of Lucas-Washburn’s law or Lucas-Washburn-like scaling is out of question. However it does not apply in many cases^4^,^5^,^11^,^12^,^13^ as stated previously. It has been determined in this paper that one of the reasons is the ignorance of gravity in Lucas-Washburn’s law. Also investigated was how and when gravity should be considered.

In summary, the criteria for using the Lucas-Washburn law has been proposed as follows. The specific value of CGR number 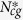 was determined to be about 3.0. The effect of gravity force on spontaneous imbibition may be ignored and Lucas-Washburn’s law applies when 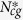 > 3.0; the effect of gravity force cannot be neglected and Equation (1) may be utilized when 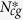 < 3.0.

The model and phenomenon described in this paper are of both fundamental and applied interest; important parameters such as effective water permeability, for example, may be inferred using the imbibition test data. With this model, one may easily conduct spontaneous imbibition experiments in a vertical direction, which obviates the need for difficult horizontal experiments that exclude the effect of gravity. The data indicate that the effect of gravity on imbibition can be considered theoretically and the model described here extended Lucas-Washburn’s law, which has been widely used in many industries and research areas for almost a century.

The number of examples for applying the results in this article is limited. There are many more areas and cases, including nanostructured materials^26^,^27^, where liquid imbibition happens and the ideas proposed in this article may be useful. Note that the imbibition rates may be much faster at nanoscale than those in porous media with conventional sizes^4^,^7^.

## Additional Information

**How to cite this article**: Li, K. *et al.* Criteria for Applying the Lucas-Washburn Law. *Sci. Rep.* **5**, 14085; doi: 10.1038/srep14085 (2015).
